# Impact of Intragranular Substructure Parameters on the Forming Limit Diagrams of Single-Phase B.C.C. Steels

**DOI:** 10.3390/ma6115217

**Published:** 2013-11-13

**Authors:** Gérald Franz, Farid Abed-Meraim, Marcel Berveiller

**Affiliations:** 1 Laboratoire des Technologies Innovantes (LTI), EA 3899—IUT Amiens, Avenue des Facultés—Le Bailly, Amiens Cedex 1 F-80025, France; E-Mail: gerald.franz@u-picardie.fr; 2 Laboratoire d’Etude des Microstructures et de Mécanique des Matériaux (LEM3), UMR CNRS 7239—Arts et Métiers ParisTech, 4 rue Augustin Fresnel, Metz Cedex 3 F-57078, France; E-Mail: farid.abed-meraim@ensam.eu; 3 Laboratory of Excellence on Design of Alloy Metals for low-mAss Structures (DAMAS), University of Lorraine, Ile du Saulcy, Metz Cedex 1 F-57045, France

**Keywords:** forming limit diagrams, bifurcation analysis, intragranular substructure, multiscale model, crystal plasticity, B.C.C. materials

## Abstract

An advanced elastic-plastic self-consistent polycrystalline model, accounting for intragranular microstructure development and evolution, is coupled with a bifurcation-based localization criterion and applied to the numerical investigation of the impact of microstructural patterns on ductility of single-phase steels. The proposed multiscale model, taking into account essential microstructural aspects, such as initial and induced textures, dislocation densities, and softening mechanisms, allows us to emphasize the relationship between intragranular microstructure of B.C.C. steels and their ductility. A qualitative study in terms of forming limit diagrams for various dislocation networks, during monotonic loading tests, is conducted in order to analyze the impact of intragranular substructure parameters on the formability of single-phase B.C.C. steels.

## 1. Introduction

In order to plastically deform thin metallic sheets into elaborate 3D shapes, forming processes, such as deep drawing, are used in the automotive industry. These processes consist of multi-stage operations, inducing strain-path changes in the material, which often result in softening/hardening effects that are observed on the stress-strain curves. These macroscopic effects may critically alter the strain distribution and consequently lead to flow localization and ultimately sheet metal fracture.

The proper modeling for the behavior of polycrystalline single-phase steels, which are commonly used in sheet metal forming processes, requires an accurate description of the most important sources of plastic anisotropy acting at different characteristic length scales, e.g., intragranular substructure evolution (microscopic scale), plastic slip processes (mesoscopic scale), and texture development (macroscopic scale).

The aim of the current paper is to investigate the effect of microstructural features on the formability of single-phase B.C.C. steels. It is nowadays widely recognized that localized necking is one of the main phenomena that restrict industrial sheet metal forming processes, and for stretched metal sheets, the associated limit strains are commonly represented using the concept of forming limit diagram (FLD), which depicts the maximum allowable strains for various loading paths. For this reason, the present work only focuses on the onset of strain localization in the form of macroscopic shear bands; other failure modes such as buckling, wrinkling, or diffuse necking are not considered.

The choice of the constitutive modeling has been shown to significantly affect the FLD predictions [1,2,3]. As most often the phenomenological constitutive framework does not account for the macroscopic softening/hardening effects induced by strain-path changes, considerable attention has been devoted in the last decades to advanced behavior models in order to improve FLD predictions. In the literature, various FLD analyses have been carried out with micromechanical models using the full-constraint Taylor scale-transition scheme (see, e.g., [4,5,6,7,8,9]). More recently, a self-consistent rate-dependent polycrystalline plasticity model has been adopted for FLD investigations [10].

In a recent study, a micromechanics-motivated multiscale model has been developed, including a detailed description of the heterogeneous dislocation distribution based on Peeters’ works [11,12]. In this previous contribution [13], the polycrystalline behavior was obtained using a self-consistent scale-transition scheme. On the other hand, in another earlier investigation [14], a formability criterion based on the loss of ellipticity of the governing boundary value problem has been coupled with micromechanical constitutive models that did not include any intragranular heterogeneity description.

Given that the main objective here is to investigate the impact of the intragranular substructure development on the FLDs, only the constitutive equations for the microscopic modeling of the advanced elastic-plastic self-consistent (EPSC) model and the selected plastic instability criterion are briefly recalled in this paper. The interested reader is referred to [13] for a comprehensive presentation of the overall multiscale model. The ability of this multiscale model to properly reproduce the evolution of the substructural features and to accurately predict the macroscopic behavior of single-phase polycrystalline steels for monotonic and sequential loading paths has already been shown in [13].

In what follows, a detailed qualitative strain localization analysis is carried out for a 1000-grain polycrystalline aggregate, with the aim of establishing relationships between the heterogeneous dislocation distribution and the overall formability—as represented by the entire FLD—of single-phase B.C.C. steels. Note that a preliminary strain localization investigation has been conducted in [15] for a similar ferritic steel; however, the analysis was restricted to the particular loading path of plane strain tension, which corresponds to the lowest point of the FLD. Here, the analysis is enlarged to the entire FLD to determine the impact of each intragranular substructure component on the overall formability.

## 2. Elastic-Plastic Self-Consistent Modeling Taking Dislocation Patterning into Account

### 2.1. Single Crystal Plasticity Modeling

Plastic anisotropy of elastic–plastic materials partly develops at the mesoscopic scale, and the principal mechanism of plastic deformation for B.C.C. and F.C.C. structures is the crystallographic slip. As the current EPSC multiscale model incorporates microscopic modeling based on experimental observations of B.C.C. structure, the application of the single crystal modeling is thus restricted to these materials. The corresponding deformation occurs by shear on the slip planes {110} and {112}, in the slip directions <111>, constituting 24 independent slip systems potentially active.

A complete presentation of the single crystal plasticity modeling providing the large-strain kinematics and the method of slip system selection is available in [14], while the local incremental elastic–plastic constitutive equations can be found in [13].

### 2.2. Intragranular Substructure Modeling

#### 2.2.1. Description of Dislocation Networks inside Grains of Deformed B.C.C. Metals

The development of heterogeneous intragranular substructure is regarded as source of plastic anisotropy occurring at the microscopic level. The microscopic modeling relies on the work of Peeters *et al.* [11,12], starting from experimental observations on B.C.C. grains.

**Figure 1:**
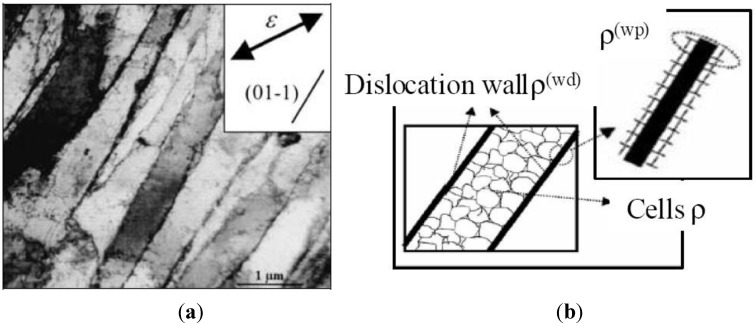
(**a**) Longitudinal plane view transmission electron microscopy (TEM) micrograph of a grain of an IF-steel (interstitial-free steel) specimen after 20% uniaxial tension in RD (after Peeters *et al.* [11]). (**b**) Schematic representation of the heterogeneous dislocation microstructure.

Dislocation networks appear in deformed B.C.C. metals after a sufficient amount of plastic deformation. These dislocation distributions have the same characteristics in all grains of the polycrystal. Their size, shape and orientation depend on the applied strain paths (monotonic or sequential). Several experimental studies [16,17,18,19,20] have revealed that dislocations are generally arranged heterogeneously; consisting of alternating continuous planar regions of high local dislocation density—the dislocation sheets—surrounding low local dislocation density zones, called the cell interiors. An example of these dislocation arrangements is shown on the transmission electron microscopy (TEM) micrograph of a grain of a single-phase ferritic steel after a tensile test in the rolling direction (RD), depicted in Figure 1a.

The heterogeneous dislocation configuration generally observed inside grains of deformed B.C.C. metals is described by three local dislocation densities, as illustrated in Figure 1b.

A single local dislocation density ρ represents the randomly stored dislocations inside cells constituting the low dislocation density areas. This density is considered as responsible for the isotropic hardening of the metallic sheet. The high local dislocation density regions can be represented by two other types of dislocation densities. The first one, denoted ρ^wd^, is assumed to produce the latent hardening of the B.C.C. metals and represents the immobile dislocations trapped in the dense dislocation sheets. The second one, denoted the local polarized dislocation density ρ^wp^, reproduces asymmetry in slip resistance thanks to the sign—or polarity—of its value, and expresses the movable dislocation pilling up on both sides of the associated dense dislocation sheet.

The evolution equations of the three above-mentioned dislocation densities, representing the internal state variables of the current model, present a similar separation between two elementary mechanisms. The immobilization process between dislocations is reproduced thanks to a hardening term, while the annihilation of dislocations is described by a softening term.

The form of these equations depends directly on the current slip activity and, therefore, it is necessary to distinguish the evolution of currently existing dislocation walls from that of previously existing dislocation sheets, which allows us to account for the effect of the pre-existing microstructure and consequently for the strain-path history of the material.

As observed experimentally on TEM micrographs of grains of deformed B.C.C. metals [19], the model will construct at most two families of dislocation sheets, parallel to the {110} planes on which the highest and second highest slip activity rates occur.

At each strain increment, the intragranular heterogeneous dislocation distribution can be defined from the knowledge of slip activity in each grain of the polycrystalline aggregate, which is given by the single crystal plasticity modeling discussed in the previous section. In Franz *et al.* [13], it has been shown that the proposed model is able to correctly reproduce the intragranular substructure for single crystals under different crystallographic orientations during various loading conditions (monotonic tests, Bauschinger shear tests, and cross tests).

#### 2.2.2. Intensity of Currently Existing Dense Dislocation Walls

The intensity of currently existing dense dislocation walls represents the value of the local immobile dislocation density ρ^wd^ associated with them. Its evolution is given by a balance equation expressed as a function of the total slip rate ${\dot{\Gamma }}_{i}$ on the crystallographic plane on which the *i^th^* greatest slip activity occurs:

(1) $${\dot{\rho }}_{i}^{\text{wd}}=\frac{1}{b}\left(I^{\text{wd}}\sqrt{\rho_{i}^{\text{wd}}}-R^{\text{wd}}\rho_{i}^{\text{wd}}\right){\dot{\Gamma }}_{i}$$

with *b* the magnitude of the Burgers vector.

During a given deformation mode, a part of mobile dislocations is stored in dense sheets generated parallel to their slip plane, in order to offer minimal resistance to the slip activity. This mechanism is expressed by the first term of Equation (1), where *I*^wd^ is a model interaction parameter, reflecting the immobilization of dislocations inside the dense walls.

After a sufficient amount of strain, corresponding to the stage III of the work-hardening curve, annihilation processes between immobile dislocations stored in the dense dislocation sheet and the mobile dislocations occur by cross slip. These processes are described by the second term of Equation (1), thanks to the model recovery parameter *R*^wd^, thus reproducing the annihilation of stored dislocations inside the dense walls.

#### 2.2.3. Polarity Assigned to Currently Existing Dense Dislocation Walls

The polarity assigned to currently existing dense dislocation walls corresponds to the accumulation of mobile dislocations of the same sign moving on the slip systems non-coplanar to these sheets and stopped on both sides of them. The sign of these mobile dislocations is opposite for the two sides of the dense dislocation sheets. The polarity of dense dislocation walls is given by the value of the local polarized dislocation density ρ^wp^ associated with them. Its evolution can be expressed in the same manner as the previous evolution law (1):

(2) $${\dot{\rho }}_{i}^{\text{wp}}=\left(\text{sign}\left(\phi_{i}^{\text{wp}}\right)I^{\text{wp}}\sqrt{\rho_{i}^{\text{wd}}+\left|\rho_{i}^{\text{wp}}\right|}-R^{\text{wp}}\rho_{i}^{\text{w}p}\right)\left|\phi_{i}^{\text{wp}}\right|$$

The net flux $\phi_{i}^{\text{wp}}=\sum_{s=1}^{n}\frac{{\dot{\gamma }}^{s}}{b}{\text{m}}^{s}\cdot {\text{n}}_{i}^{w}$ of the moving dislocations from all the slip systems non-coplanar to the *i^th^* currently existing dense dislocation wall is calculated thanks to the slip rate ${\dot{\gamma }}^{s}$ on slip system *s*, whose value can be positive or negative in order to account for the different slip directions on a particular slip system. The slip system *s* is characterized by its unit slip direction vector ${\text{m}}^{s}$, and a total of *n* slip systems (*n* = 24 for B.C.C. crystals) is considered. The currently existing dense dislocation wall *i* is represented by its unit normal vector ${\text{n}}_{i}^{w}$.

The first part of Equation (2) assumes that the creation of polarized dislocations is due to the interaction processes between the mobile dislocations and either immobile dislocations stored in the dense dislocation walls or movable dislocations pilled-up at the boundary of these walls. This term is associated with the model immobilization parameter *I*^wp^ reflecting the stopping of mobile dislocations moving on slip systems non-coplanar to a dense dislocation wall.

The recovery processes take place only between mobile dislocations and polarized dislocations of the opposite sign, as expressed by the second term of Equation (2) with the model recovery parameter *R*^wp^.

In the particular case of reversed dislocation flux, the evolution law of polarity assigned to currently existing dense dislocation walls is only given by a recovery term, expressed thanks to the model recovery parameter *R*_rev_, as follows:

(3) $${\dot{\rho }}_{i}^{wp}=-R_{\text{rev}}\rho_{i}^{\text{wp}}\left|\phi_{i}^{\text{wp}}\right|$$

The net flux $\phi_{i}^{\text{wp}}$ contributing to a dense dislocation sheet *i* can be positive or negative. The change of its sign is possible during a reverse test. Slip activity occurs on the same slip systems but in the opposite sense. Thus, the polarity dislocations that have already been stuck along dense sheets, due to the prestrain loading, can easily move away and be annihilated by dislocations of opposite sign.

#### 2.2.4. Randomly Stored Dislocations inside Cells

The randomly stored dislocations inside cells are assumed to be responsible for the isotropic hardening of the deformed B.C.C. materials. The evolution of this local density can be expressed as the summation of two elementary terms: the hardening one with the model immobilization parameter *I* and the softening one with the model recovery parameter *R*. Furthermore, it was experimentally observed that the local density of the randomly stored dislocations in the cell interiors tends to decrease during load reversal, because of the activation, in the opposite sense, of most of the slip systems that were previously active. In order to account for this phenomenon, an additional term of annihilation can be activated with a binary switch parameter Ψ depending on the reversal of the net flux $\phi_{i}^{\text{wp}}$ associated with a family *i* of currently existing walls. The importance of this supplementary annihilation process is quantified by the model recovery parameter *R*_2_. Thus, the evolution law of the density of randomly stored dislocations can be expressed as follows:

(4) $$\dot{\rho }=\frac{1}{b}\langle \left(I\sqrt{\rho }-R\rho \right)\sum_{s=1}^{n}\left|{\dot{\gamma }}^{s}\right|-\text{Ψ}R_{2}\frac{\rho^{\mathrm{bausch}}}{2\rho_{\mathrm{sat}}^{\mathrm{wp}}}\sum_{s=1}^{n}\left|{\dot{\gamma }}^{s}\right|\rangle$$

where 〈y〉=y if y>0 and 〈y〉=0 otherwise. The value of $\rho^{\mathrm{bausch}}$ will differ depending on the number of reversed fluxes.

#### 2.2.5. Previously Existing Dense Dislocation Walls

Dislocation networks currently observed in deformed B.C.C crystals are associated with current slip activity. When other slip systems are activated, consecutively to strain-path changes or because of the rotation of a grain towards a stable orientation, new families of dense dislocation walls corresponding to the current strain path will be, thus, formed and, at the same time, the previously generated dislocation sheets will be also disintegrated. The evolution of the two local densities associated with previously existing dense dislocation walls is given thanks to a model recovery parameter *R*_ncg_ characterizing the destruction of former dense dislocation sheets:

(5) $${\dot{\rho }}_{i}^{\text{wd}}=-\frac{R_{\text{ncg}}}{b}\rho_{i}^{\text{wd}}{\dot{\Gamma }}_{\text{new}},\;{\dot{\rho }}_{i}^{wp}=-\frac{R_{\text{ncg}}}{b}\rho_{i}^{\text{wp}}{\dot{\Gamma }}_{\text{new}}$$

where ${\dot{\Gamma }}_{\text{new}}$ is the total slip rate on both of the crystallographic planes containing the highest slip activity.

#### 2.2.6. Critical Resolved Shear Stresses

As shown above, the contributions of isotropic hardening, latent hardening and polarity are related to the three internal state variables of the present model. Following Mughrabi’s two-phase composite model [21], the dense dislocation walls can be considered as the hard phase, due to their high dislocation density, while the randomly stored dislocations inside cells can represent the soft phase with low dislocation density. The resulting critical resolved shear stress on each slip system *s* can be expressed as the summation of these three contributions as follows:

(6) $$\tau_{c}^{s}=\tau_{c0}^{s}+\left(1-f\right)\alpha \mu b\sqrt{\rho }+f\sum_{i=1}^{6}\alpha \mu b\left(\sqrt{\rho_{i}^{\text{wd}}}\left|{\text{m}}^{s}\cdot {\text{n}}_{i}^{w}\right|+\langle \sqrt{\left|\rho_{i}^{\text{wp}}\right|}\left({\text{m}}^{s}\cdot {\text{n}}_{i}^{w}\right)\text{sign}\left(\rho_{i}^{\text{wp}}\right)\rangle \right)$$

where *f* is the volume fraction of the dislocation sheets, α the dislocation interaction parameter, µ the shear modulus, and $\tau_{c0}$ the initial critical resolved shear stress.

### 2.3. Scale Transition Scheme—Macroscopic Stress/Strain Response of Single-Phase B.C.C. Steel

Single-phase polycrystalline aggregates feature an initial crystallographic texture that evolves during loading with the grains rotating towards preferred stable orientations. Such reorientations are responsible, at the macroscopic scale, for the anisotropic behavior of the deformed B.C.C. metals.

At each strain increment, the behavior of the individual constituents of the polycrystalline aggregate is known from the single crystal plasticity modeling. In order to derive the overall macroscopic response of polycrystalline aggregates from the results of single crystals, the self-consistent scheme in the sense of Hill [22] is adopted. Such an averaging approach allows accounting for the morphological and crystallographic evolutions for each grain during loading. For the numerical implementation of the single crystal constitutive equations, the fourth-order Runge–Kutta algorithm is used, which is known to be more robust and reliable than the forward Euler time integration scheme. As this explicit scheme is conditionally stable, the selected size for the loading increments is kept sufficiently small to preserve both stability and accuracy all along the simulation of the entire loading paths. The self-consistent scheme, which is strongly implicit by nature, is solved using a classical fast convergence iterative algorithm. In this process, an enhanced inversion technique is introduced to deal with ill-conditioned matrices that are encountered in the course of loading, which allows us to simulate the entire loading paths with improved accuracy. A complete presentation of this averaging approach is given in [14], along with the full details on the numerical implementation of the local constitutive law, as well as the computational aspects relating to the self-consistent scale-transition scheme.

**Figure 2:**
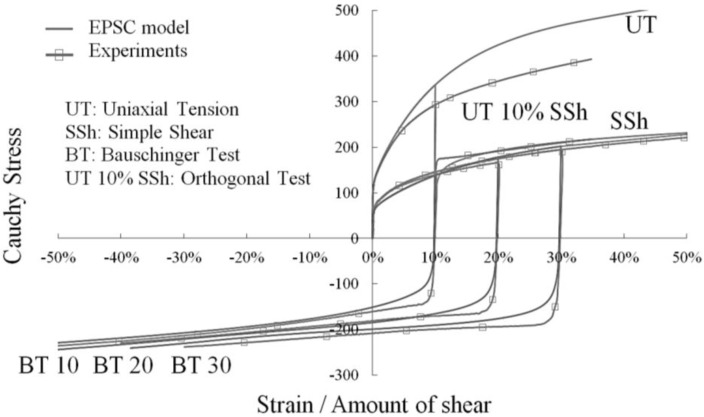
Comparison model/experiments for the stress–strain response of an IF–Ti steel along various strain paths performed parallel to RD.

**Figure 3:**
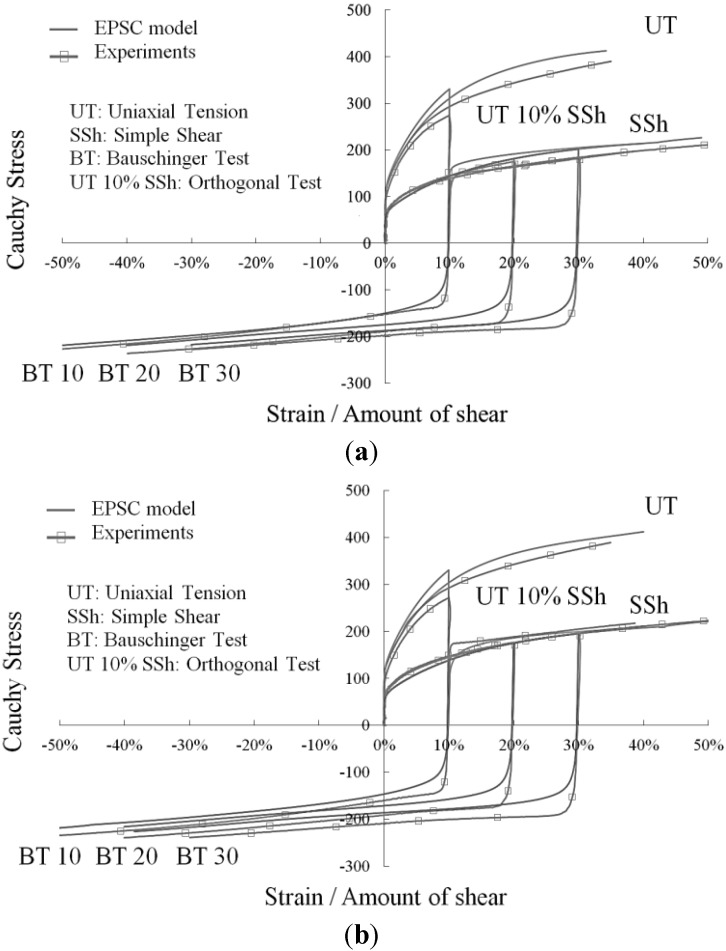
Comparison model/experiments for the stress–strain response of an IF–Ti steel along various strain paths performed: (**a**) at 45° with respect to RD, (**b**) at 90° with respect to RD.

As depicted in Figure 2 and Figure 3a,b, the macroscopic stress–strain curves obtained with the proposed model, for different loading directions with respect to RD, are in reasonable agreement with experimental tests for an IF–Ti ferritic single-phase steel. The model parameters for this steel are identified according to the procedure detailed in [13], and are reported below in Table 1. Note that these material parameters correspond to a value α = 0.48 for the dislocation interaction parameter that enters Equation (6). The obtained simulation results, along with other validation tests reported elsewhere, demonstrate the ability of this advanced multiscale model to reproduce the elastic–plastic behavior of single-phase polycrystalline materials.

**Table 1:** Material model parameters identified for an interstitial-free (IF)–Ti steel.

***I***	***R* (m)**	***I*^wd^**	***R*^wd^ (m)**	***I*^wp^**	***R*^wp^ (m)**
1.8 × 10^−2^	2 × 10^−9^	6.6 × 10^−2^	1.9 × 10^−9^	2.9 × 10^−2^	2 × 10^−8^
***R*_ncg_ (m)**	***R*_rev_ (m)**	***R*_2_ (m)**	***f***	**τ_*c*0_(110) (MPa)**	**τ_*c*0_(112) (MPa)**
1 × 10^−10^	3 × 10^−8^	5 × 10^−8^	0.2	45	45

## 3. Ductility Loss Modeling

### 3.1. Rice’s Localization Criterion

Two distinct approaches are generally used in the literature to investigate plastic instability: on the one hand, engineering methods based on experimental or empirical observations and, on the other hand, sound theoretical approaches using either bifurcation or stability theories.

**Figure 4:**
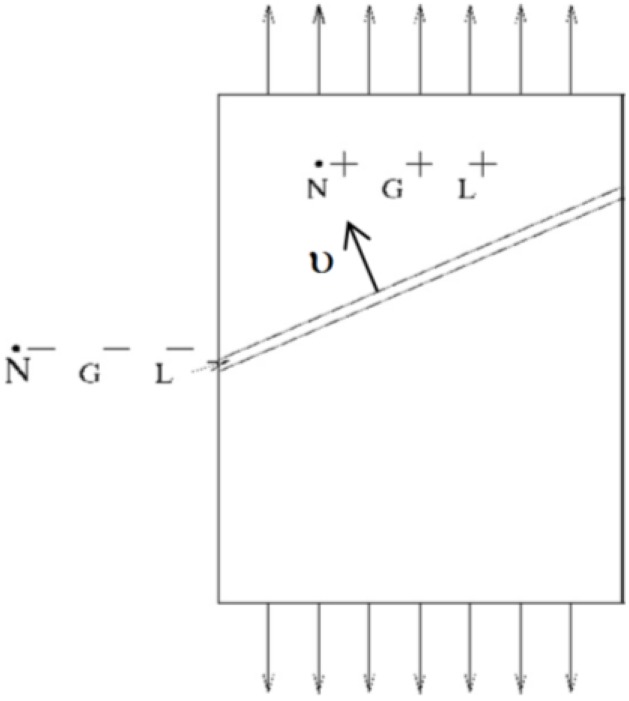
Illustration of the localization of the deformation along a shear band: mechanical fields outside the band are denoted by superscript +.

The criterion selected in this study belongs to the second category; *i.e.*, the so-called Rudnicki–Rice criterion [23,24], which corresponds to a bifurcation associated with admissible jumps for strain and stress rates across a shear band, as illustrated in Figure 4. It is noteworthy that an important difference with the Marciniak–Kuczynski [25] localization approach, which postulates a pre-existing initial defect in the form of a localization band of reduced thickness in the metal sheet, is that the bifurcation theory does not need any arbitrary used-defined fitting parameter. Another advantage with this intrinsic bifurcation criterion, besides its sound theoretical foundations, is that it allows a fully three-dimensional localization analysis and thus the determination of the out-of-plane orientation of the localization band, contrary to most analyses that assume plane stress conditions.

Because field equations have to be satisfied, and because the velocity gradient is discontinuous across the localization band, a kinematic condition for the strain rate jump must be verified. In addition, the continuity of the stress rate vector has to be verified for the forces along the interface created by the localization band. For more details, the interested reader may refer to the pioneering contributions by Rice and coworkers [23,24].

The combination of all of these conditions results in a localization criterion, which will be used as a ductility limit indicator. The obtained criterion corresponds to the singularity of the acoustic tensor, and can be interpreted as loss of ellipticity of the partial differential equations governing the associated boundary value problem. It is easily expressed as a function of the only macroscopic elastic–plastic tangent modulus **L**, for the polycrystalline aggregate, and the unit vector **υ**, normal to the localization band (see Figure 4), which reads:

(7) det(υ⋅L⋅υ)=0

### 3.2. Forming Limit Diagram for Single-Phase B.C.C. Steel

The FLD for the IF–Ti ferritic single-phase steel, for which the model parameters are reported in Table 1, is predicted using the proposed advanced EPSC approach coupled with the above-discussed bifurcation criterion.

In order to assess the predictive capability of the present model in the determination of forming limit strains, it is necessary to compare our results with a reference FLD. The FLD provided by ArcelorMittal is obtained using a model developed by Cayssials [26]. The latter can be considered as reference for comparison because it has proven its reliability in predicting formability for linear strain paths for a wide range of grades of sheet metals, for which experimental FLDs have been simultaneously measured and compared.

**Figure 5:**
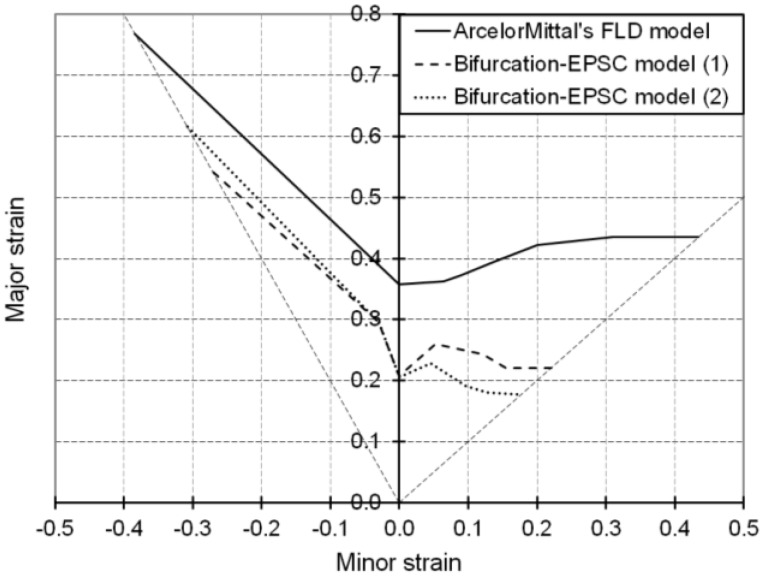
Simulated FLDs associated with linear strain paths for the IF–Ti single-phase steel as obtained with Bifurcation–EPSC and ArcelorMittal’s models. For the Bifurcation–EPSC model, (1) corresponds to α = 0.48 and (2) to α = 0.2.

As shown in Figure 5, the FLDs obtained with the Bifurcation–EPSC model for the studied ferritic single-phase steel have shapes similar to that of ArcelorMittal’s FLD, although they fall somewhat lower. It is noteworthy that for the EPSC model, two values for the dislocation interaction parameter α [see Equation (6)] can be found in the literature [27,28] for B.C.C. materials. These two values, which will be denoted in Figure 5 as (1) for α = 0.48 and (2) for α = 0.2, result inherently in two different sets of material parameters after identification. In our previous study, the value α = 0.2 was taken and the corresponding identified material parameters are provided in [15]. We investigate here the effect of this dislocation interaction parameter by considering the value α = 0.48, for which the associated identified material parameters are given in Table 1. Note that this latter value of α, which is taken in the current study, is motivated by physical interpretations and micromechanics-based calculations (see reference [27]), and thus more commonly adopted in the literature [29]. From Figure 5, it appears that an increase in this parameter has no significant impact on the ductility limit for monotonic strain paths ranging from uniaxial tension to plane strain tension. A lower value of α leads, however, to earlier strain localization in the biaxial expansion domain of the FLD.

In what follows, attention will be focused on the investigation of the impact of substructure features on formability limits of single-phase B.C.C. materials. Some of the underlying motivation in such an attempt of establishing relationships between microstructural properties and material ductility is to provide a prediction tool able to classify materials in terms of ductility and, at longer term, to optimize material properties or to design new grades of steel with enhanced in-use mechanical properties.

## 4. Impact of Substructure Features on the Forming Limit Strains of Single-Phase B.C.C. Steel

The qualitative analysis of the impact of microstructural mechanisms, related to model parameters, on ductility is conducted on a 1000-grain polycrystalline aggregate similar to the ferritic single-phase steel IF–Ti, for which the identified parameter values are reported in Table 1. The results are shown using linear FLDs plotted for monotonic loading paths ranging from uniaxial tension to equibiaxial expansion. Due to the negligible influence of the parameters that are specific to sequential loading tests (*i.e.*, *R*_ncg_, *R*_rev_, and *R*_2_) on the forming limit strains during monotonic tests, only the model parameters associated with the cell interiors and intensity of dense dislocation sheets will be analyzed in what follows. In this process, only one parameter is varied at a time, in order to investigate its effect on ductility, while all of the remaining model parameters are kept constant.

### 4.1. Impact of the Randomly Distributed Dislocation Network

**Figure 6:**
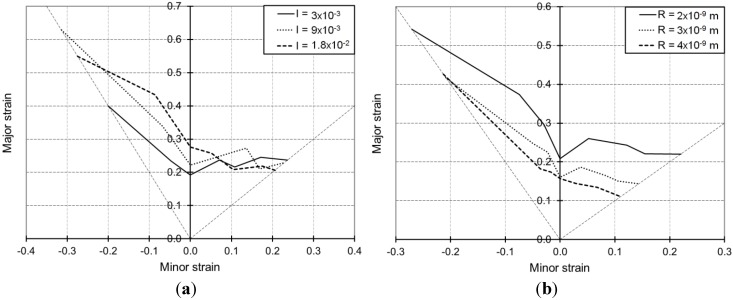
Simulated FLDs associated with linear loading paths for the IF–Ti single-phase steel as obtained with the Bifurcation–EPSC model: Effect of the model parameters associated with the randomly distributed dislocation network.

Figure 6a,b is obtained by varying the immobilization parameter *I* and the recovery parameter *R*, respectively, while keeping all the remaining parameters of Table 1 constant. The evolution of randomly distributed dislocations is given by Equation (4) in the modeling at the microscale. As explained previously, this equation contains two opposite terms representing, respectively, the storage and annihilation mechanisms for the dislocations randomly distributed in the cell interiors.

The proportion of dislocations that are going to be captured inside cells is defined by the immobilization parameter *I*. Thus, larger values for this parameter induce more obstacles to slip and consequently an increase in hardening leading to better ductility. This trend is disclosed by the proposed model, in the whole, as shown in Figure 6a.

In the same way, the recovery parameter *R* reflects the significance of the annihilation of randomly distributed dislocations. Thus, larger values for this parameter denote a more important quantity of dislocations that are going to disappear, making slip motion easier and consequently an overall softening of the material promoting early strain localization. This trend is very well reflected by the proposed model, as shown in Figure 6b.

The selected values for parameter *I*, respectively *R*, have been chosen to represent a reasonable range of variation (*i.e.*, 2.5 × 10^12^, 9 × 10^13^ and 2.5 × 10^12^, 2 × 10^13^·m^−2^, respectively) for the local saturated dislocation density for the cells, corresponding to a stationary cell structure. Such saturation values for the randomly distributed dislocation density are easily obtained by equating the right-hand side of Equation (4) to zero, which corresponds to the stagnation of the cell structure. Larger values for parameter *I* induce higher values for the local saturated dislocation density of the cells, whereas larger values for parameter *R* allow reaching faster this saturation stage.

### 4.2. Impact of the Intensity of Dense Dislocation Sheets

**Figure 7:**
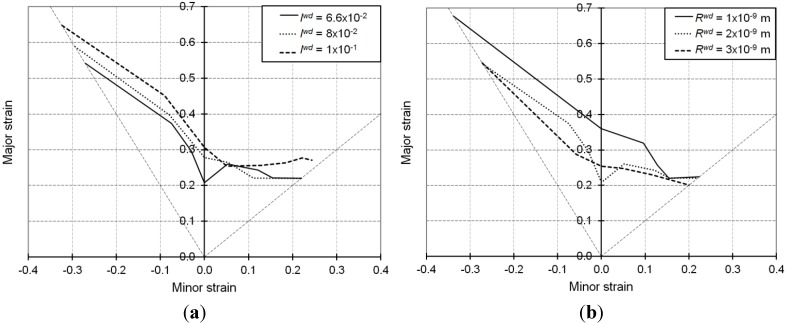
Simulated FLDs associated with linear loading paths for the IF–Ti single-phase steel as obtained with the Bifurcation–EPSC model: Effect of the model parameters associated with the intensity of dense dislocation sheets.

Figure 7a,b is obtained by varying the immobilization parameter *I*^wd^ and the recovery parameter *R*^wd^, respectively, while the remaining model parameters of Table 1 are kept constant.

These two parameters *I*^wd^ and *R*^wd^ reflect the same elementary mechanisms as parameters *I* and *R*, respectively; however, they pertain to the dislocations stuck in the dense dislocation sheets.

The evolution of the local immobile dislocation density associated with the dense dislocation sheets is expressed by Equation (1) in the microscopic modeling. Similarly to Equation (4), this equation distinguishes the storage and annihilation mechanisms for the dislocations stored in the walls using two terms with opposite sign.

Larger values for the immobilization parameter *I*^wd^ produce dense dislocation walls with higher intensity. As discussed in the previous section, the dense dislocation sheets are generated parallel to the crystallographic planes on which the slip activity is greatest. Consequently, there will be fewer dislocations acting as obstacles to the slip of other dislocations, which leads to increased ductility as shown in Figure 7a.

With the same way of reasoning as for parameter *R* discussed previously, the recovery parameter *R*^wd^ leads to comparable effects. Accordingly, larger values for this parameter tend to promote earlier strain localization as shown by the overall level of the FLDs plotted in Figure 7b.

The selected values for parameter *I*^wd^, respectively *R*^wd^, have been chosen to represent a reasonable range of variation (between 4.84 × 10^14^ and 2.77 × 10^15^ m^−2^) for the local saturated dislocation density for the dense walls. Such saturation values for the local immobile dislocation density associated with the dense dislocation sheets are obtained by equating the right-hand side of Equation (1) to zero, which corresponds to the impossibility of storing dislocations in the walls any further. Larger values for parameter *I*^wd^ induce higher values for the immobile dislocation density associated with the dense dislocation sheets, whereas larger values for parameter *R^w^*^d^ allow reaching faster the saturation of this dislocation density.

### 4.3. Impact of the Volume Fraction of Dense Dislocation Sheets

We investigate here the influence of the volume fraction *f* of dense dislocation sheets by varying this parameter while keeping constant all of the parameters in Table 1.

**Figure 8:**
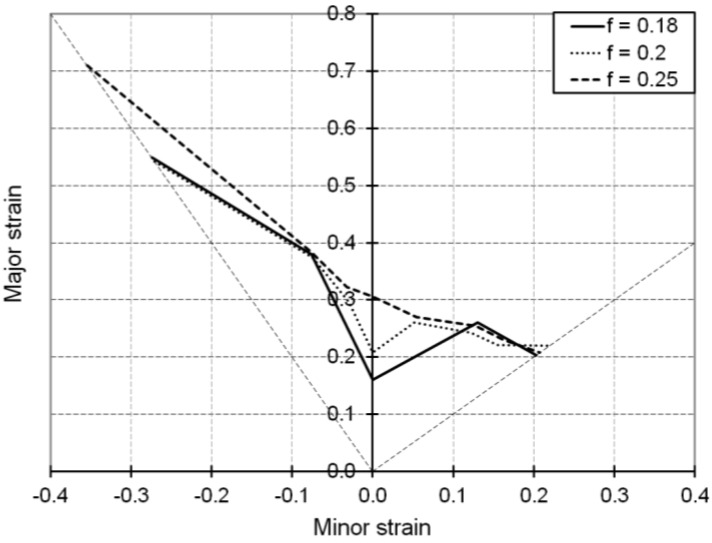
Simulated FLDs associated with linear loading paths for the IF–Ti single-phase steel as obtained with the Bifurcation–EPSC model: Effect of the volume fraction *f* of dense dislocation sheets.

As suggested by Figure 8, increasing the volume fraction *f* of dense dislocation sheets tends to improve the overall ductility of B.C.C. metals. This effect is in agreement with the influence of the immobilization parameter associated with dense dislocation walls, because increasing the presence of dislocation walls has an effect that is somehow similar to increasing their intensity.

### 4.4. Impact of the Initial Critical Resolved Shear Stress

Finally, a last analysis in this parameter sensitivity study concerns the effect of the initial critical resolved shear stress $\tau_{c0}$, by assigning different values to this parameter while keeping constant all of the remaining parameters in Table 1.

**Figure 9:**
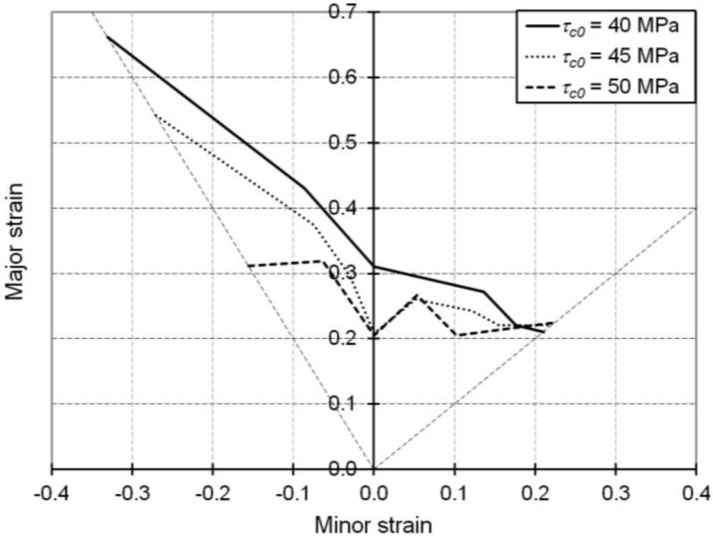
Simulated FLDs associated with linear loading paths for the IF–Ti single-phase steel as obtained with the Bifurcation–EPSC model: Effect of the initial critical resolved shear stress $\tau_{c0}$.

As shown in Figure 9, decreasing values for the initial critical resolved shear stress, and by extension those associated with the elastic limit, lead to improved overall ductility for single-phase B.C.C. metals. This effect is in agreement with the work of Luft [30], who reported that for single crystals of molybdenum solicited in uniaxial tension, a decrease in temperature resulted in an increase in the elastic limit and, thus, a drop in ductility.

## 5. Conclusions

In this paper, the bifurcation-based localization criterion, first proposed by Rice, has been coupled with an advanced elastic-plastic self-consistent multiscale model. The latter incorporates microscopic modeling with an elaborate description of the development and evolution of intragranular substructure of single-phase B.C.C. steels.

Numerical FLDs have been first determined for a ferritic single-phase steel, denoted IF–Ti, and compared to a reference FLD provided by ArcelorMittal. In this process, the macroscopic behavior law has been accurately modeled in order to take into account the most important microstructural aspects, *i.e.*, initial and induced textures, dislocation densities and softening mechanisms. The proposed EPSC multiscale model has proven its ability to correctly reproduce the stress–strain responses for various mechanical tests (linear as well as sequential two-stage strain paths), and at different loading directions with respect to the rolling direction.

Then, qualitative numerical investigations have been conducted by varying different model parameters in order to identify the impact of the intragranular dislocation network, *i.e.*, the dislocations stored inside cells and the dense dislocation sheets, on the formability of single-phase B.C.C. steels. The current investigation enlarges our previous preliminary study [15], which was restricted to the only plane strain tension loading path, by extending the analysis to the entire set of strain paths that are required to determine the complete FLD. The most influential parameters revealed in the preliminary analysis are confirmed as well as the general trends regarding their impact on overall ductility.

The obtained results also demonstrate the capability of the proposed theoretical and numerical tool to predict and compare the formability of B.C.C. materials and may serve for hierarchical classification of metals with regard to ductility. Therefore, it could be used to optimize the ductility of new steels or to design materials with desired formability and in-use properties.
